# Associations of overweight/obesity with patient-reported outcome measures after oblique lumbar interbody fusion

**DOI:** 10.3389/fsurg.2024.1360982

**Published:** 2024-06-20

**Authors:** Lan-Li Hsueh, Yun-Che Wu, Chien-Chou Pan, Cheng-Min Shih, Cheng-Hung Lee, Jun-Sing Wang, Kun-Hui Chen

**Affiliations:** ^1^ Department of Orthopedics, Taichung Veterans General Hospital, Taichung, Taiwan; ^2^ Department of Rehabilitation Science, Jenteh Junior College of Medicine, Nursing and Management, Miaoli, Taiwan; ^3^ Department of Physical Therapy, Hungkuang University, Taichung, Taiwan; ^4^ Department of Post-Baccalaureate Medicine, College of Medicine, National Chung Hsing University, Taichung, Taiwan; ^5^ Department of Food Science and Technology, Hung Kuang University, Taichung, Taiwan; ^6^ Division of Endocrinology and Metabolism, Department of Internal Medicine, Taichung Veterans General Hospital, Taichung, Taiwan; ^7^ Department of Computer Science and Information Engineering, Providence University, Taichung, Taiwan

**Keywords:** oblique lateral interbody fusion (OLIF), overweight, obesity, clinical outcome, spinal fusion

## Abstract

**Background:**

Oblique lateral interbody fusion (OLIF) combined with transpedicular screw fixation has been practiced for degenerative spinal diseases of elderly patients for years. However, overweight patients have been shown to have longer operative times and more complications from surgery. The effect on clinical outcome is still uncertified. The objective of this study was to determine is overweight a risk factor to clinical outcome of OLIF combined with transpedicular screw fixation technique.

**Material and methods:**

A retrospective study in patients submitted to OLIF combined with transpedicular screw fixation from January 2018 to August 2019 was conducted. VAS score, ODI score and EQ5D were measured before the operation and one year after the operation.

**Results:**

A total of 111 patients were included with 48 patients in the non-obese group and 55 patients in the overweight/obese group. There was no significant difference between the two groups in gender, age, smoking history, hypertension, chronic kidney disease and diabetes mellitus. Overweight/obese group has higher BMI (28.4 vs. 22.7, *p* < 0.001) than non-obese group. There was no difference between the two groups in pre-operative VAS score, ODI score and EQ5D score. However, the healthy weight group improved much more than the overweight score in VAS score, ODI score and EQ5D score.

**Conclusion:**

The overweight/obese patient group had clinical outcomes worse than the non-obese group in terms of pain relief and life functions.

## Introduction

Degenerative lumbar spine disease affecting 2.4%–5.7% of population world-wide, is an important cause of disability and poor life quality (1). In addition to normal aging, several risk factors of non-communicable diseases have been associated with degenerative spinal diseases and its perioperative complications, such as obesity (2–6). According to the World Health Organization, obesity is now a major public health issue. In 2016, more than 1.9 billion adults of 18 years and older were overweight. Of these more than 650 million were obese. In the United States, obese people have incurred medical care costs from 68.4% to 233.6% of those with normal weight (7).

Oblique lumbar interbody fusion (OLIF) was a surgical procedure first described by Mayer in 1997 (8). OLIF represents a relatively minimal invasive lumbar interbody fusion, adopting an approach from spine anterior to the psoas, instead of using a transpsoas approach. This approach is different from the traditional approach. Therefore, during OLIF, no adhesion from the previous surgery is encountered, increasing the perioperative safety of the procedure. During OLIF procedure, the patient is placed in a lateral position, with abdominal contents shifted to the contralateral side, basically away from the incision wound, vertebral body and disc. Obesity likely lengthens operation time and increases blood loss in transforaminal lumbar interbody fusion (TLIF) and minimally invasive TLIF (MIS-TLIF), but not in the case of OLIF. OLIF hence markedly reduces additional costs associated with performing lumbar fusions on obese patients (9).

However, no clear evidence is available on the relationship between obesity and clinical outcomes after OLIF surgery. Here, we aimed to study the associations between obesity and patient-reported outcome measures after OLIF procedure in the Taiwanese population.

## Methods

All patients provided written informed consent, after the institutional review board of the hospital had approved our study (approval number: CE22167A). We enrolled patients who had undergone OLIF for symptomatic spinal stenosis or spondylolisthesis from January 2018 to August 2019. OLIF was performed after the diagnosis of degenerative spinal diseases that included spondylolisthesis with segmental instability, severe spinal stenosis, disc herniation or other degenerative disease were confirmed. Patients had received conservative care, including physical therapy, anti-inflammatory medications, and injection therapy for more than three months but in vain without improvement.

Only patient aged between 18 and 80 years old were enrolled. We excluded those who had undergone OLIF but without posterior fixation, with a follow-up period of ≤6 months, those non Taiwanese patients. From January 2018 to August 2019, a total of 126 patients underwent OLIF surgery. Among them, 3 patients were non-Taiwanese, 9 patients were over 80 years old, 3 patients received OLIF without PI and 8 patients did not complete follow-up. Therefore, a total of 103 patients were finally included. In the end, we collected fata from 103 patients in terms of their age, gender, height, weight, preoperative diagnosis, operation level, smoking history, systemic diseases including diabetes and hypertension, previous operation history including spinal surgery and abdominal surgery history.

Level of overweight/obesity were determined based on WHO's classification that included the following categories: (a) underweight or normal weight: BMI <25 kg/m^2^; (b) overweight: BMI 25–29.9 kg/m^2^; (c) obese: BMI 30–39.9 kg/m^2^; and (d) severe obese: BMI ≥40 kg/m^2^. In our study, we created only two broad categories: (a) non-obese group, i.e., BMI <25 kg/m^2^, and (b) overweight/obese group which means BMI ≥25 kg/m^2^ (10, 11).

### Surgical technique

Under general anesthesia, the patient was put on the right lateral decubitus position, sterilized and dressed according to standard procedure. A 6-cm oblique incision was made anterior to the center of the disc. The external oblique, internal oblique and transverse fascia were dissected. The retroperitoneal space was subsequently retracted anteriorly to expose the anterior corridor of the psoas muscle. The level of disc was confirmed from the scanogram. Guided pin and serial dilators were inserted, and a retractor was applied parallelly to the disc space and fixed with a pin screw. Discectomy and end-plate preparation was made. Then the serial cage trials were checked. A Medtronic Clydesdale cage was filled with the demineralized bone matrix. The three layers of muscle were repaired and wound closed layer by layer. The patient was then turned to the prone position. A midline incision was made. Nerve decompression was performed as the surgeon's decision. Transpedicular screws system was subsequently applied free-handed or with robotic-assisted placement of pedicle screw. The wound was finally closed layer by layer.

### Patient-reported outcome measures

Patient-reported outcome measures included the visual analog scale (VAS) (12) for back and dominant leg pain, Oswestry disability index (ODI) (13), and EuroQol- 5 Dimension (EQ-5D) (14). The questionnaires were completed first on the day before the operation at ward and later at one month, 3 months and 6 months after the operation.

### Statistical analyses

Differences of continuous and categorical variables between non-obesity (BMI <25 kg/m^2^) and overweight/obese groups (BMI ≥25 kg/m^2^) were compared using independent t test and chi-square test, respectively. The associations of BMI ≥25 kg/m^2^ (vs. BMI <25 kg/m^2^) with changes from baseline to 6-month follow-up in patient-reported outcome measures were examined using linear regression analyses with multivariate adjustment. Statistically significance difference was set at *p *< 0.05. All the statistical analyses were conducted using the Statistical Package for the Social Sciences (IBM SPSS version 22.0; International Business Machines Corp., NY, USA).

## Results

**Figure 1:**
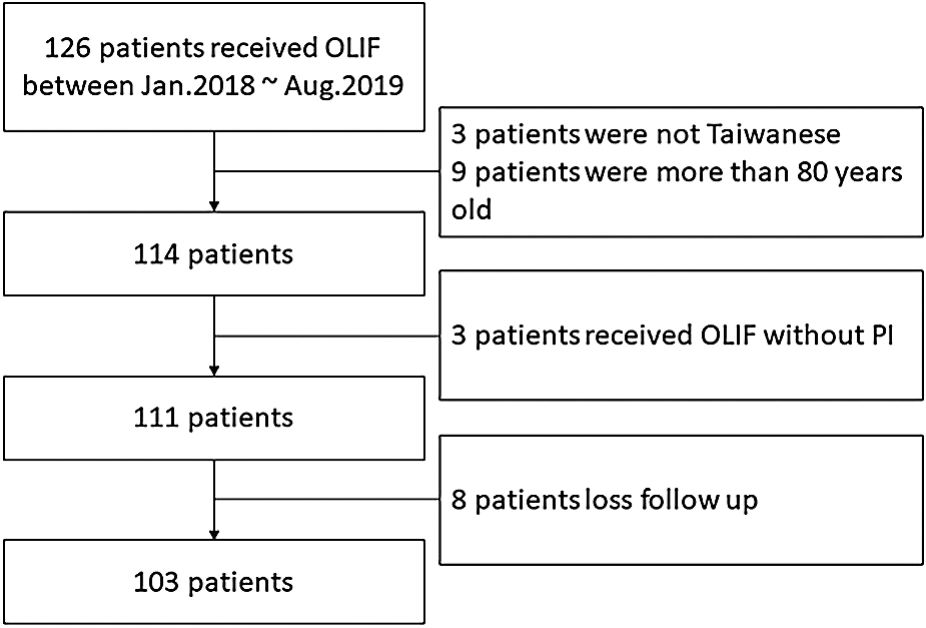
Flowchart of patient identification.

Of the 111 OLIF patients who were initially enrolled, 8 patients lost follow-up and were excluded from the analysis (Figure 1). None of these patients underwent the surgery for non-degenerative diseases (tumor or trauma). A total of 103 patients (35 males and 68 females) who met inclusion criteria were finally analyzed. There were 48 patients in the non-obese group and 55 patients in the overweight/obese group. Their mean BMI was 22.7 ± 1.6 kg/m^2^ in the non-obese group, and 28.4 ± 2.6 kg/m^2^ in the overweight/obese group with significant difference between the two groups (*p < *0.001). On the other hand, no such significant difference was found in terms of their age (*p *= 0.253), gender (*p *= 0.335), smoking history (*p *= 0.404), diabetes mellitus (*p *= 0.847), hypertension (*p *= 0.192), and abdominal surgery history (*p *= 0.576). In the non-obese group, 8 patients (16.7%) patients received OLIF for adjacent segment disease (ASD) and 7 patients (12.7%) in the overweight/obese group received OLIF for ASD. The proportions of revision surgery of the two groups were similar. The number of involved levels was 2.9 ± 1.1 in the non-obese group and 3.0 ± 1.1 in the overweight/obese group (*p *= 0.582) (Table 1).

**Table 1:** Baseline characteristics of the study population according to body mass index.

Variables	Non-obesity <25 kg/m^2^	Overweight/Obesity ≥25 kg/m^2^	*p*-value
*N*	48	55	
Age, years	64.3 ± 8.0	62.3 ± 9.7	0.253
Male sex, *n* (%)	14 (29.2)	21 (38.2)	0.335
Body mass index, kg/m^2^	22.7 ± 1.6	28.4 ± 2.6	<0.001
Smoking, *n* (%)	3 (6.3)	6 (10.9)	0.404
Diabetes, *n* (%)	14 (29.2)	17 (30.9)	0.847
Hypertension, *n* (%)	20 (41.7)	30 (54.5)	0.192
Chronic kidney disease, *n* (%)	10 (20.8)	8 (14.5)	0.402
History of abdominal surgery, *n* (%)	6 (12.5)	5 (9.1)	0.576
Number of vertebral body involved	2.9 ± 1.1	3.0 ± 1.1	0.582
Distribution of spine level involved			0.388
Single-level	23 (47.9%)	19 (34.5%)	
2-level	15 (31.3%)	15 (27.3%)	
≥3-level	10 (20.8%)	21 (38.2%)	
Surgical duration, min	447 ± 105	440 ± 118	0.757
Blood loss, ml	436 ± 115	436 ± 118	0.994

Values are mean ± SD or *n* (%).

In the non-obesity patients, 23 (47.9%) patients received single-level surgery, 15 (31.3%) patients received two-level surgery and 10 (20.8%) patients received more than three-level surgery. In the obesity patients, 19 (34.5%) patients received single-level surgery, 15 (27.3%) patients received two-level surgery and 21 (38.2%) patients received more than three-level surgery. There was no significant between-group difference in the distribution (*p *= 0.388). The operation time and intraoperative blood loss are listed in Table 1 showing similar findings for the non-obese and the overweight/obese groups. The operation time (447 ± 105 min vs. 440 ± 118 min, *p *= 0.757) and intraoperative blood loss (436 ± 115 ml vs. 436 ± 118 ml, *p *= 0.994) were comparable between the two groups.

Clinical outcomes for the non-obese and overweight/obese groups are shown in Table 2 according to the times before surgery, one month and 6 months after surgery. Pain level, as assessed through VAS scores, was similar in the two groups before surgery. However, at one month after surgery, the VAS score was significantly lower in the non-obese group (*p *= 0.026). The variations of VAS scores between pre-surgery and at 1 month or 6 months post-surgery were significantly different between the two groups.

**Table 2:** Changes in patient-reported outcome measures according to body mass index.

Variables	<25 kg/m^2^	≥25 kg/m^2^	*p*-value
Visual analog scale for pain			
Baseline	8.1 ± 1.4	7.8 ± 1.2	0.327
1 month after OLIF	3.7 ± 1.7	4.4 ± 1.5	0.026
Change from baseline to 1 month	−4.4 ± 1.7	−3.5 ± 1.6	0.033
6 months after OLIF	2.7 ± 2.1	3.3 ± 2.1	0.142
Change from baseline to 6 months	−5.4 ± 2.2	−4.5 ± 2.1	0.033
Oswestry disability index			
Baseline	55.9 ± 9.4	53.6 ± 8.8	0.202
1 month after OLIF	46.6 ± 10.2	48.4 ± 9.7	0.349
Change from baseline to 1 month	−9.2 ± 10.9	−5.2 ± 8.9	0.036
6 months after OLIF	30.7 ± 15.1	33.3 ± 16.0	0.401
Change from baseline to 6 months	−25.4 ± 13.7	−20.0 ± 13.5	0.046
EQ-5D			
Baseline	0.35 ± 0.09	0.37 ± 0.06	0.243
1 month after OLIF	0.59 ± 0.07	0.57 ± 0.07	0.077
Change from baseline to 1 month	0.24 ± 0.11	0.20 ± 0.08	0.029
6 months after OLIF	0.69 ± 0.12	0.65 ± 0.12	0.125
Change from baseline to 6 months	0.33 ± 0.15	0.28 ± 0.12	0.043

Values are mean ± SD. EQ-5D, EuroQol-5D; OLIF, oblique lateral interbody fusion.

The ODI scores were similar between the two groups at all the three time points: i.e., before surgery, one month and 6 months after surgery. The non-obese patients showed greater improvement in ODI scores after surgery than the overweight/obese patients both at one month (−9.2 ± 10.9 vs. −5.2 ± 8.9, *p *= 0.036) and 6 months (−25.4 ± 13.7 vs. −20.0 ± 13.5, *p *= 0.046).

Almost the same trend was found for the EQ-5D scores. EQ-5D scores were similar between the 2 groups before surgery, one month and 6 months after surgery. The variation of EQ-5D scores between pre-surgery and 6 months post-surgery was statistically significant (*p *= 0.043).

Different models were applied to analyze the associations of BMI with changes in patient-reported outcome measures and were shown in Table 3. Model 1 showed the higher BMI, the less improvement in VAS scores, the ODI scores and the EQ-5D scores, and without any adjustment. Model 2 showed the same trend after adjusting with age and gender. Model 3 showed the same result after adjusting effects for age, gender, smoking, diabetes, hypertension, chronic kidney disease, history of abdominal surgery, number of vertebral bodies involved, and surgical duration. These models showed that the higher BMI was associated with the lesser improvements in patient-reported outcome measures after OLIF surgery.

**Table 3:** Associations of body mass index with changes in patient-reported outcome measures.

Body mass index ≥25 kg/m^2^ vs. <25 kg/m^2^	β coefficient (95% CI)	*p*-value
Changes from baseline to 6 months in VAS for pain		
Model 1	0.910 (0.074, 1.746)	0.033
Model 2	0.967 (0.118, 1.816)	0.026
Model 3	0.950 (0.080, 1.820)	0.033
Changes from baseline to 6 months in ODI		
Model 1	5.417 (0.086, 10.748)	0.046
Model 2	6.814 (1.741, 11.886)	0.009
Model 3	6.486 (1.386, 11.586)	0.013
Changes from baseline to 6 months in EQ-5D		
Model 1	−0.054 (−0.106, −0.002)	0.043
Model 2	−0.068 (−0.117, −0.019)	0.007
Model 3	−0.064 (−0.115, −0.013)	0.014

Model 1, unadjusted. Model 2, adjusted for age and sex. Model 3, adjusted for variables in Model 2 plus smoking, diabetes, hypertension, chronic kidney disease, history of abdominal surgery, number of vertebral body involved, and surgical duration. EQ-5D, EuroQol-5D; ODI, oswestry disability index; VAS, visual analog scale.

## Discussions

Obesity is known to be highly correlated with spinal degeneration diseases including spinal stenosis, disc herniation and spondylolisthesis (3, 5). With the increasing prevalence of obesity, the relationship between obesity and efficacy of spinal surgery has become more important. However, the definition of obesity in mostly western studies is BMI >30 kg/m^2^, which is not directly applicable to Asians. Our study is the first of its kind in exploring the effects between obesity and spine in Taiwanese patients using the cutoff point of BMI >25 kg/m^2^, a standard which is more appropriate for Asian populations (15).

In our study, both overweight/obese and non-obese patients showed significant improvements after the spine surgery. When patients were followed up at 6 months after surgery, non-obese patients showed greater improvement than overweight/obese patients. Whether patients were divided into two groups based on BMI (less than 25 kg/m^2^ and greater than 25 kg/m^2^) or into three groups based on BMI (less than 25 kg/m^2^, between 25 kg/m^2^ and 30 kg/m^2^, and greater than 30 kg/m^2^), the same trend can be observed. Non-obese patients showed greater improvement in those patient-reported outcome measures than overweight/obese patients.

Both VAS pain scores and functional scores (EQ-5D and ODI) revealed the same trend in a retrospective review of 271 patients. Djurasovic et al. found no difference in mean improvements between overweight/obese and non-obese patients based on Short Form-36 (SF-36) physical composite summary and ODI (6). An as-treated analysis was performed on patients treated for lumbar disc herniation, as they were enrolled in the Spine Patient Outcomes Research Trial (SPORT). The trail results revealed that overweight/obese patients who had been managed operatively had significantly smaller improvements than non-obese patients in terms of SF-36 and ODI (4). In our study, the obese group showed slightly poorer clinical performance before surgery, and significantly less clinical improvement after the surgery. This finding was similar to the aforementioned researches. On the other hand, a systematic review showed inconsistent effects of obesity on clinical outcomes in spine intervention (16). The relationship between obesity and clinical outcomes of spine surgery remains unclear. Obesity increases the difficulty of spinal surgery. However, due to the lateral decubitus position during OLIF, the abdominal fat tissue shifts away from the surgical site, thereby reducing the difficulty of the surgery of interbody fusion. Previous studies did not focus on the relationship between clinical outcomes and obesity in OLIF surgery. To the best of our knowledge, this study is the first to address this issue.

In 2010, Meredith et al. retrospectively reviewed 75 cases on one or two level lumbar microdiscectomy from L2-S1 performed by a single surgeon with a minimum follow-up period of 6 months. They found that obesity was associated with a 12 fold higher chance of postoperative recurrent herniation, and a 30 fold chance of requiring reoperation (17). Kim et al. also reported a similar association between BMI and recurrence following percutaneous endoscopic lumbar discectomy. Patients with recurrent lumbar disc herniation have higher BMIs compared with those without recurrence, and their mean symptom-free interval is 2.5 (range: 0.5–27) months (18). In our study, the cage subsidence rate [43/48 (89.6%) vs. 51/55 (92.7%), *p *= 0.230], screw loosening rate [3/48 (6.3%) vs. 5/55 (9.1%), *p *= 0.534] and fusion rate [43/48 (89.6%) vs. 51/55 (92.7%), *p *= 0.230] were similar between the two groups. The source of back and leg pain could be difficult to identify. More soft tissue dissection could cause more discomfort after the surgery. In an obese patient, lumbar extensor muscles must work even harder to support their higher body weight. Recurrent disc herniation after surgery appeared to be an important issue for obese patients with HIVD. Dietary behaviors and dietary quality can lead to chronic inflammation and affect chronic musculoskeletal pain conditions (19). The source of back and leg pain could be difficult to identify. There is a need for more pathology-specific studies in humans focusing on specific musculoskeletal pain conditions and underlying pain-generation mechanisms.

OLIF is often associated with postoperative anterior thigh pain on the approach side due to the antepsoas approach and psoas major muscle retraction to insert the interbody cage orthogonally. A prospective study collected and analyzed the anterior thigh pain and associated factors following OLIF. Sixty-five of the total patients (70.6%) experienced approach-side anterior thigh pain to any extent during postoperative 0–7 days following OLIF. The mean pain VAS (4.4 ± 2.1) and the prevalence (57.6%) were highest at postoperative 2 days. On postoperative day 7, there were 19 patients (20.7%) who complained of residual anterior thigh pain with a mean VAS of 2.6 ± 1.8 (20). In our hospital, the patient was put on the right lateral decubitus position and approached through the left side. Some of the patients complained of left leg pain before the surgery. However, left leg pain improved a lot after the surgery. There was no significant difference in VAS between the non-obese group and obese group before the surgery (4.6 ± 3.5 vs. 5.0 ± 3.7, *p *= 0.603), one month after the surgery (1.9 ± 2.2 vs. 2.3 ± 2.3, *p *= 0.299) and six months after the surgery (1.3 ± 2.0 vs. 1.6 ± 2.1, *p *= 0.472).

What seemed to be worse in spine operation for obese patients, when compared with non-obese patients, is their higher complication rates as reported in the literature. These include longer operative times, greater blood loss, and a higher risk of surgical site infection (21). Higher BMI causes more blood loss and longer operation time in TLIF but not in OLIF or MIS-TLIF (9). In our study, obese patients had similar operation time and similar intraoperative blood loss when compared with to non-obese patients. Such a correlation of obesity and complications were also reported in the posterior approach. Specifically, the complication rate is 14% with a BMI of 25, 20% with a BMI of 30, and 36% with a BMI of 40 (18). Following OLIF, obesity and morbid obesity in general are not associated with worse operative time, blood loss, approach-related sequelae, or complications (22).

MIS approach with percutaneous screws is indeed a good method for posterior instrumentation. The benefit of MIS approach included smaller skin incisions, less blood loss from surgery, reduced risk of muscle damage, reduced risk of infection and postoperative pain and faster recovery from surgery and less rehabilitation required (23). However, the National Health Insurance policy in Taiwan does not cover the MIS screw, which lead to a significant difference in price between minimally invasive and traditional surgical methods. The cost effectiveness of MIS spine surgery remained unknown. Factors that may increase the incremental cost effectiveness for MIS over conventional open procedures include decreased complications, shorter length of hospital stay, and faster return to work, homemaking, and productivity (24).

## Limitations

First, this is a retrospective, single-institution study. Second, for spine surgery, 6-months of follow-up might be relatively short. A short term follow-up may have confounded our findings, including the clinical improvement, fusion rate or the reoperation rate. Third, no image data was available to confirm our correctness in alignment. OLIF is better in restoring spinal alignment than TLIF. However, lack of imaging data prevents us from understanding whether the advantages are the same for obese patients as they are for patients with normal weight. This issue deserves further investigation.

## Conclusion

Patients with overweight/obesity (BMI ≥25.0 kg/m^2^) had less improvement in several patient-reported outcome measures after OLIF for degenerative spinal diseases than those who had a normal weight (BMI <25.0 kg/m^2^).

## Data Availability

The raw data supporting the conclusions of this article will be made available by the authors, without undue reservation.
